# Hypoxia Activated EGFR Signaling Induces Epithelial to Mesenchymal Transition (EMT)

**DOI:** 10.1371/journal.pone.0049766

**Published:** 2012-11-21

**Authors:** Ashish Misra, Chhiti Pandey, Siu Kwan Sze, Thirumaran Thanabalu

**Affiliations:** School of Biological Sciences, Nanyang Technological University, Singapore, Singapore; Nanyang Technological University, Singapore

## Abstract

Metastasis is a multi-step process which requires the conversion of polarized epithelial cells to mesenchymal cells, Epithelial–Mesenchymal Transition (EMT). EMT is essential during embryonic morphogenesis and has been implicated in the progression of primary tumors towards metastasis. Hypoxia is known to induce EMT; however the molecular mechanism is still poorly understood. Using the A431 epithelial cancer cell line, we show that cells grown under hypoxic conditions migrated faster than cells grown under normal oxygen environment. Cells grown under hypoxia showed reduced adhesion to the extracellular matrix (ECM) probably due to reduced number of Vinculin patches. Growth under hypoxic conditions also led to down regulation of E-cadherin and up regulation of vimentin expression. The increased motility of cells grown under hypoxia could be due to redistribution of Rac1 to the plasma membrane as opposed to increased expression of Rac1. EGF (Epidermal Growth Factor) is a known inducer of EMT and growth of A431 cells in the absence of oxygen led to increased expression of EGFR (EGF Receptor). Treatment of A431 cells with EGF led to reduced cell adhesion to ECM, increased cell motility and other EMT characteristics. Furthermore, this transition was blocked by the monoclonal antibody Cetuximab. Cetuximab also blocked the hypoxia-induced EMT suggesting that cell growth under hypoxic conditions led to activation of EGFR signaling and induction of EMT phenotype.

## Introduction

Cancer will overtake heart disease as the world's top killer by 2010 (World Health Organization) and the majority of human tumors arise from epithelial tissues (carcinomas) [1]. The ability of cancer to spread, or metastasize, is responsible for the majority of deaths associated with cancer [2]. For cancer cells to metastasize, they must detach from the primary tumor, invade and migrate into surrounding connective tissue, blood and lymphatic vessels [3]. Invasion of cancer cells is induced by the chemo-attractants secreted by other cell types [4], [5]. Epithelial cells are polarized cells and cellular transitions are crucial during the developmental stages of multicellular organisms which is apparent during gastrulation when the process of EMT transforms polarized epithelial cells into migratory mesenchymal cells [6]. The transition to mesenchymal cells gives rise to a morphology that is suitable for migration [7]. Mesenchymal cells can also revert back to epithelial cells by MET (Mesenchymal-Epithelial Transition) [6]. It is now widely established that EMT is exploited during disease states such as metastasis [8].

During EMT, epithelial cells detach from their neighbors, the underlying basement membrane, become more motile and migratory [9], [10]. The loss of epithelial phenotype and gain of mesenchyme-like phenotype are critical steps in the conversion of malignant carcinoma to invasive carcinoma and metastasis. The importance of EMT in metastasis has led to intensive investigations into the molecular mechanism in the activation of the EMT pathway which is characterized by the loss of cell-cell adhesion, repression of E-Cadherin and increased cell motility [11], [12]. In cultured cells, EMT can be induced either by physiological (eg. growth factors) or by environmental factors (eg. hypoxia) [9].

Oxygen supply is crucial for the growth of cells and is often diminished in solid tumors, especially at the centre of the tumor mass, as tumor cells grow faster than the endothelial cells that are crucial for the formation of blood vessels [13]. Normal tissues typically have median [O_2_] in the range of 40–60 mm Hg due to an efficient network of capillaries, while solid tumors have a median [O_2_] value of 10 mm Hg [14], [15]. Although hypoxia kills most normal and cancer cells, it also provides a strong selective pressure for the survival of the most aggressive and metastatic cells [16]. Thus, hypoxia in solid tumors leads to resistance to many anticancer drugs and, importantly, may accelerate malignant progression by increasing metastasis [17], [18].

Recently, we observed that A431 epithelial carcinoma cells grown under hypoxic conditions exhibited increased invasiveness and secreted factors with increased chorioallantoic membrane angiogenic activity [12]. To further understand the molecular mechanism underlying the conversion of epithelial to mesenchymal phenotypes under hypoxia, we grew the A431 cells under hypoxic conditions and characterized the molecular mechanism involved in EMT. The cells grown under hypoxic conditions lost cell-cell adhesion mediated by E-Cadherin due to down-regulation of E-Cadherin and activation of Snail [12]. The cells grown under hypoxic conditions were also more motile compared to cells grown under normal O_2_ concentrations. The increased motility is consistent with the reduced cell-ECM (Fibronectin or Collagen-I) adhesion observed. Hypoxic conditions also led to increased membrane localization of Rac1, a small molecular GTPase known to promote cell motility; however there was no increase in the expression of Rac1 suggesting that the hypoxic conditions enhanced the localization of Rac1 to the plasma membrane. Hypoxia also caused upregulation of EGFR expression and treatment of A431 cells with EGF produced phenotypic changes similar to the cells grown under hypoxic conditions. The drug Cetuximab (mono clonal antibody against the extracellular domain of EGFR) blocked EGF- and hypoxia-induced phenotypic changes. Thus our data suggests that epithelial cells grown under hypoxic conditions undergo EMT through activation of the EGFR signaling pathway.

**Figure 1:**
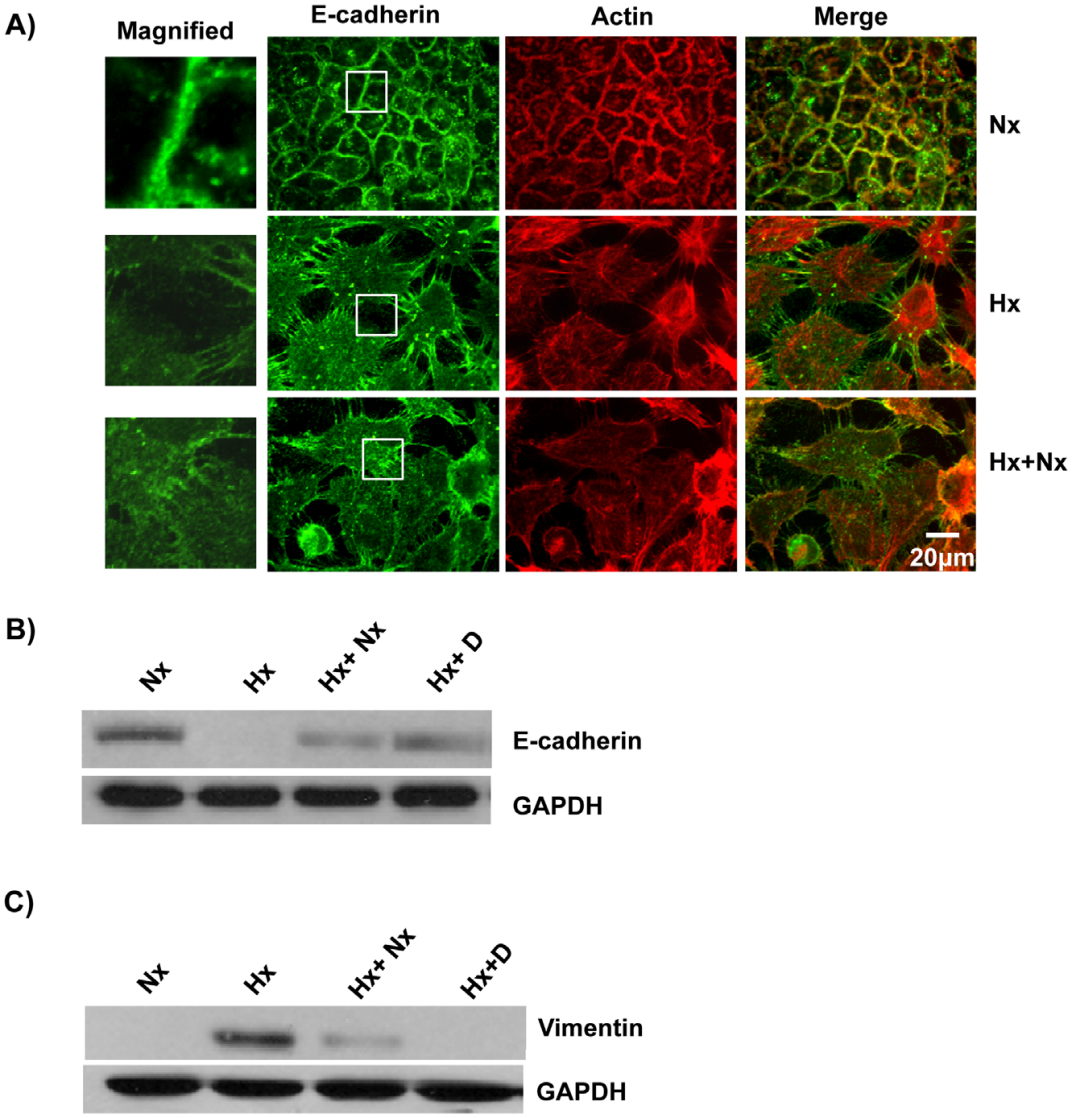
Oxygen deprivation leads to loss of Cell-Cell Adhesion. A) E-cadherin co-localized with the actin cytoskeleton in cells grown under normoxia conditions. A431 cells were grown in normoxia (Nx), hypoxia (Hx) or hypoxia+normoxia (Hx+Nx). The cells were fixed, permeabilized and probed with anti-E-cadherin followed by labeled secondary antibody (Green). The actin cytoskeleton was visualized using Alexa568-Phalloidin (Red). B) Expression of E-cadherin was reduced in cells grown under hypoxic conditions. Cell lysate from A431 cells grown under normoxia (Nx), hypoxia (Hx), hypoxia followed by normoxia (Hx+Nx) or hypoxia+Cetuximab (Hx+D) were analyzed by immunoblotting with either anti-E-cadherin or anti-GAPDH (loading control) primary antibody. C) Expression of vimentin was increased in cells grown under hypoxic conditions. Cell lysate from A431 cells grown in panel B were analyzed by immunoblotting with either anti-vimentin or anti-GAPDH primary antibody.

## Materials and Methods

### Cell Culture

A431 cell lines (ATCC: CRL-1555) were maintained in culture DMEM supplemented with 10% FBS at 37°C in a 5% CO_2_ environment. For growth under normoxia (Nx) cells were kept in 21% O_2_/5% CO_2_ for 72 h and for growth under hypoxia (Hx) conditions cells were kept under 95% N_2_/5% CO_2_ in the Modular Incubator Chamber (5352414 Billups-rethenberg). For hypoxia/reoxygenation (Hx/Nx) conditions, cells were grown under hypoxia conditions for 72 h followed 24 h under 21% O_2_/5% CO_2_. The cells were treated for 72 h with EGF (50 ng/ml) [12] and to block EMT with Cetuximab the cells were incubated with cetuximab (50 µg/ml) for the duration of hypoxic or EGF treatment.

**Figure 2:**
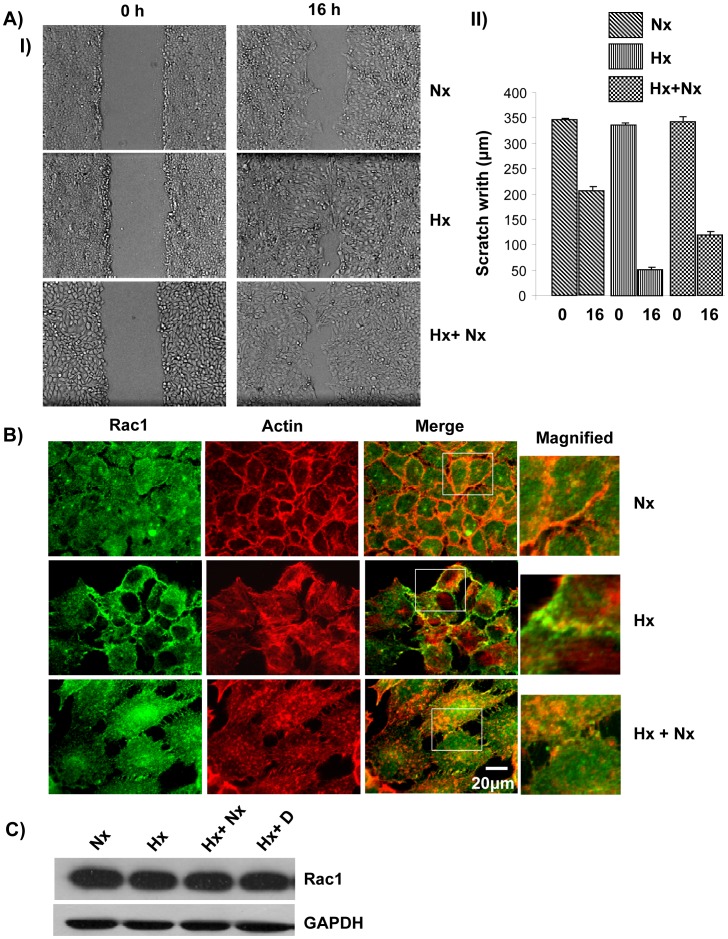
Growth under hypoxic conditions led to increased cell motility and re-localization of Rac1. A) Cells grown under hypoxic conditions displayed increased cell motility. (I) A431 cells grown under normoxia, hypoxia or hypoxia+normoxia were seeded in a 6 well dish and allowed to form a monolayer. A wound was generated and imaged at 0 h and at 16 h. (II) The width of the scratch was quantified at 10 points along the scratch and averaged. The experiment was repeated three times. B) Rac1 re-localized to the plasma membrane under hypoxic condition. A431 cells grown in normoxia, hypoxia or hypoxia+normoxia conditions were probed with anti-Rac1 followed by labeled secondary antibody (Green). The actin cytoskeleton was visualized using Alexa568-Phalloidin (Red). C) Hypoxia does not lead to increased expression of Rac1. Cell lysate from A431 cells grown under normoxia (Nx), hypoxia (Hx), hypoxia followed by normoxia (Hx+Nx) or hypoxia+Cetuximab (Hx+D) were analyzed by immunoblotting with anti-Rac1 or anti-GAPDH primary antibody.

### Cell Binding Assay

Cells were trypsinized, allowed to recover for half an hour and labeled with Calcein AM for 30 min at 37°C. Cells were then washed and re-suspended in serum-free DMEM. 2×10^5^ cells were added into each well and incubated for 25 min. Total fluorescence in each well was read in a fluorescence reader with absorption/emission wavelengths as 494/517 nm. The non-adherent cells were washed off and the fluorescence recorded (bound). The percentage of bound cells was calculated using bound fluorescence and total fluorescence [19].

**Figure 3:**
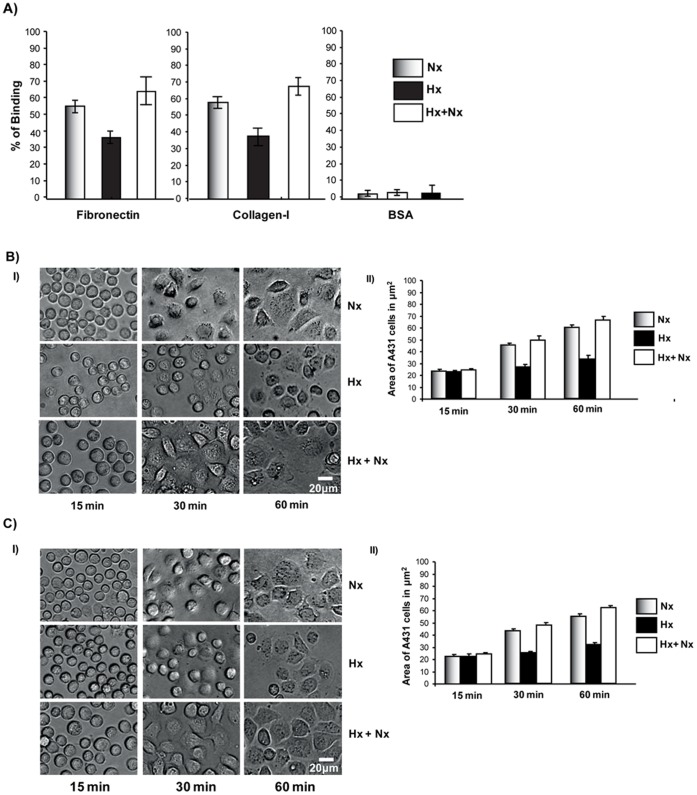
Hypoxia caused reduced cell-ECM adhesion and spreading. A) Cell-ECM adhesion is diminished in cells grown under hypoxic conditions. A431 cells were grown in normoxia, hypoxia or hypoxia+normoxia. The cells were analyzed for their cell adhesion properties as described in the Materials and Methods. B) Growth under hypoxic conditions led to decreased spreading on collagen-I. (I) A431 cells were grown in normoxia, hypoxia or hypoxia+normoxia conditions. The cells were trypsinized and plated onto coverslips coated with collagen-I. The morphology of the cells was recorded at various time intervals. (II) The surface area of the cells was measured and plotted. C) Growth under hypoxic conditions led to decreased spreading on fibronectin. (I) A431 cells were grown in normoxia, hypoxia or hypoxia+normoxia conditions. The cells were trypsinized and plated onto coverslips coated with fibronectin. The morphology of the cells was recorded at various time intervals. (II) The surface area was measured and plotted.

### Cell Spreading Assay

Cells were seeded on ECM protein coated 96-well plates at a density of 2×10^5^cells/ml and incubated at 37°C in a humidified incubator containing 5% CO_2_. Samples were viewed at 10 min, 30 min, and 60 min time intervals. The assays were performed in triplicate and the error bars represent the S.D. of 3 independent experiments. Total 90 cells were quantified for surface area for each well. The mean surface area and number of cells were calculated by using MetaMorph software.

**Figure 4:**
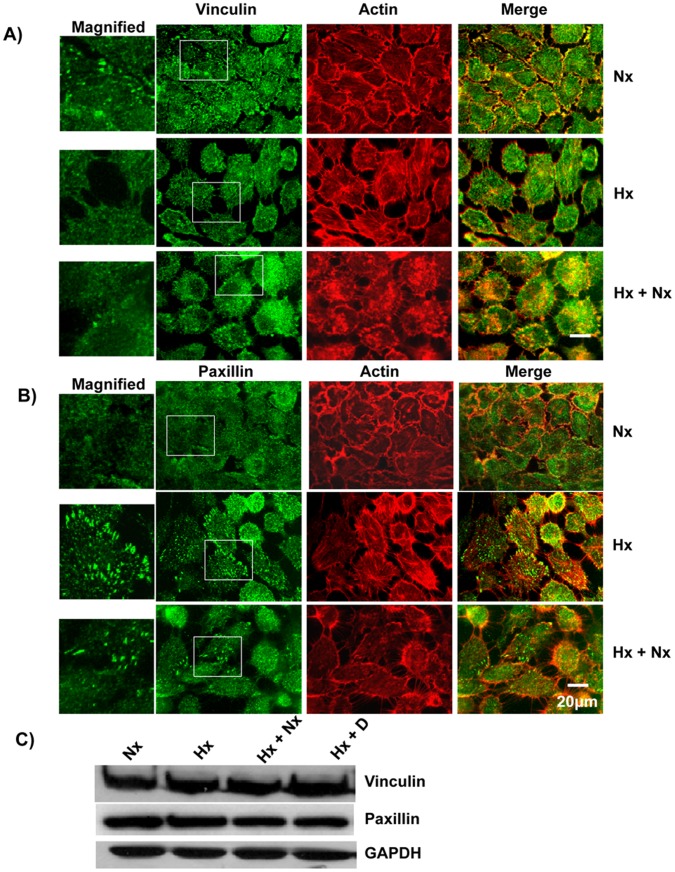
Hypoxia caused redistribution of vinculin and paxillin. A) Hypoxia led to reduction of vinculin patches. A431 cells were grown in normoxia, hypoxia or hypoxia+normoxia conditions and probed with anti-vinculin primary antibodies followed by labeled secondary antibodies (Green). The actin cytoskeleton was visualized using Alexa568-Phalloidin (Red). B) Hypoxia led to an increase in paxillin patches. A431 cells grown under normoxia, hypoxia or hypoxia+normoxia conditions were fixed, permeabilized and probed with anti-Paxillin followed by labeled secondary antibody (Green). The actin cytoskeleton was visualized using Alexa568-Phalloidin (Red). C) Hypoxia does not alter the expression of vinculin or paxillin. Cell lysate from A431 cells grown under normoxia (Nx), hypoxia (Hx), hypoxia followed by normoxia (Hx+Nx) or hypoxia+Cetuximab (Hx+D) were analyzed by immunoblotting with anti-paxillin or anti-GAPDH primary antibody.

### Scratch Assay

Cells were grown in DMEM under appropriate conditions till a monolayer was formed. A wound was generated by creating a scratch using a sterile 200 µl pipette tip. Images were acquired immediately after the scratch at 0 h and after 16 h incubation at 37°C.

**Figure 5:**
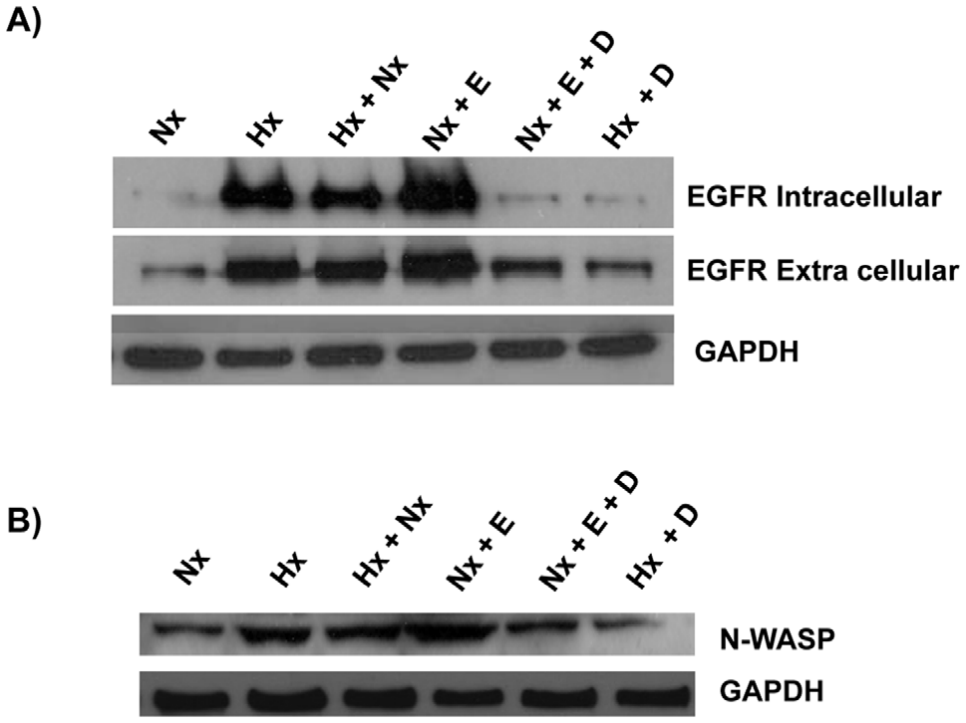
Hypoxia induced increased expression of EGFR and N-WASP. A) A431 cells were grown under the following conditions; normoxia (Nx), hypoxia (Hx), hypoxia+normoxia (Hx+Nx), normoxia+EGF (Nx+E), normoxia+EGF+Cetuximab (Nx+E+D) or hypoxia+Cetuximab (Hx+D). Cell lysate was prepared and analyzed by immunoblotting with anti-EGFR (extracellular or intracellular) primary antibodies or anti-GAPDH primary antibody. B) The A431 cell lysate prepared as described in panel A was analyzed by immunoblotting with anti-N-WASP primary antibodies or anti-GAPDH primary antibody.

### Immunoblotting

Cells were lysed using RIPA lysis buffer, the resulting lysate (30 µg of protein) was boiled in a SDS-PAGE sample buffer for 5 mins, resolved on a 10% SDS-PAGE gel and transferred onto nitrocellulose membrane. The membrane was probed with appropriate primary antibody and secondary antibody conjugated with horse radish peroxidase (HRP). Extra-cellular domain Anti-EGFR, mAb, MA1-37605, Intra-cellular rabbit mAb MA1-25877 (Thermo Scientific).

**Figure 6:**
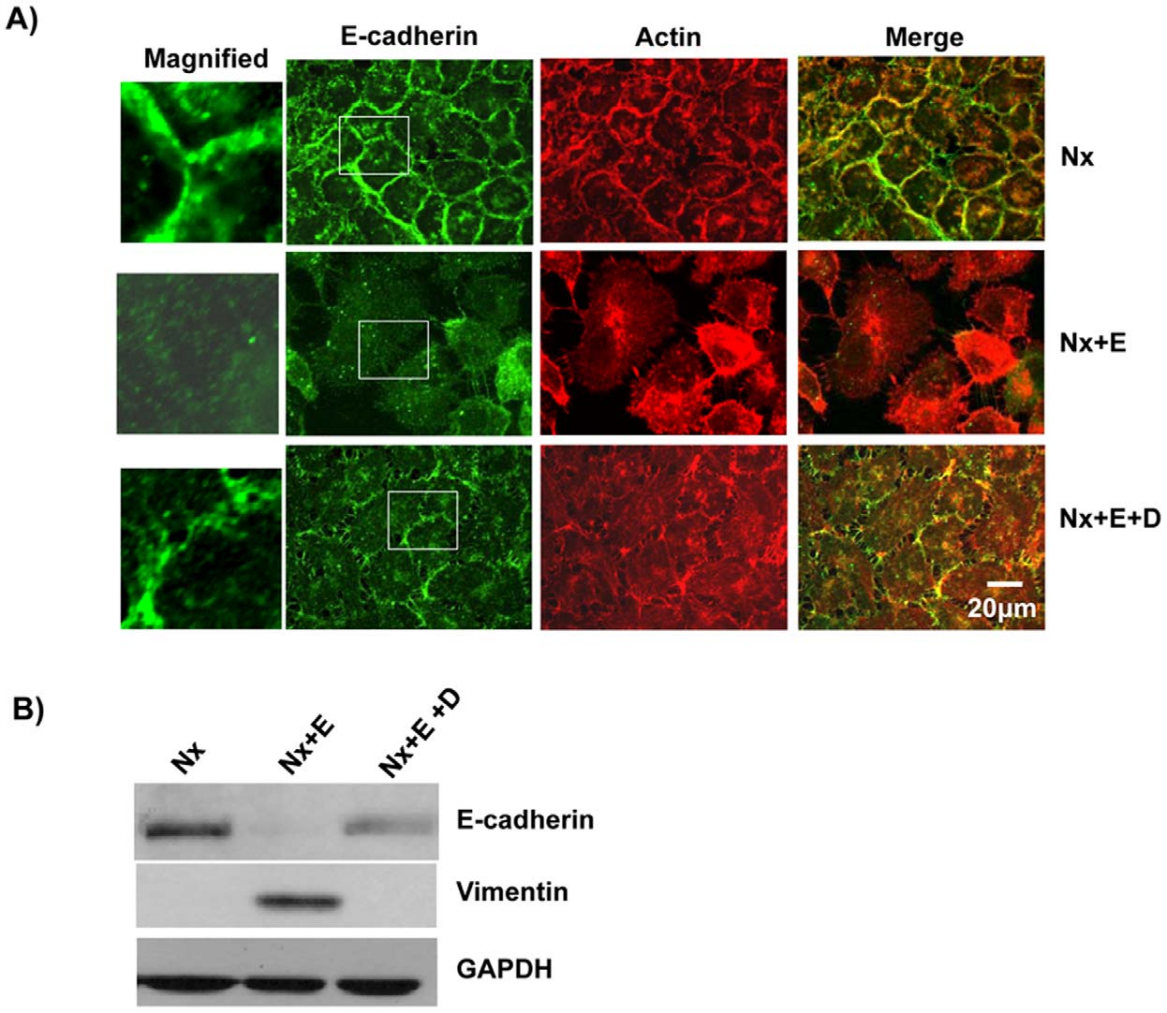
EGF treatment led to loss of E-cadherin expression and increased expression of Vimentin. A) EGF treatment leads to loss of cell-cell contacts. A431 cells were grown in normoxia (Nx), normoxia+EGF (Nx+E) or normoxia+EGF+Cetuximab (Nx+E+D). The cells were fixed, permeabilized and probed with anti-E-cadherin followed by labeled secondary antibodies. The actin cytoskeleton was visualized using Alexa568-Phalloidin. B) Cetuximab blocked the EGF induced alteration of vimentin and E-cadherin expression. Cell lysate from A431 cells grown as in panel A were analyzed by immunoblotting with anti-vimentin, anti-E-cadherin or anti-GAPDH primary antibody.

**Figure 7:**
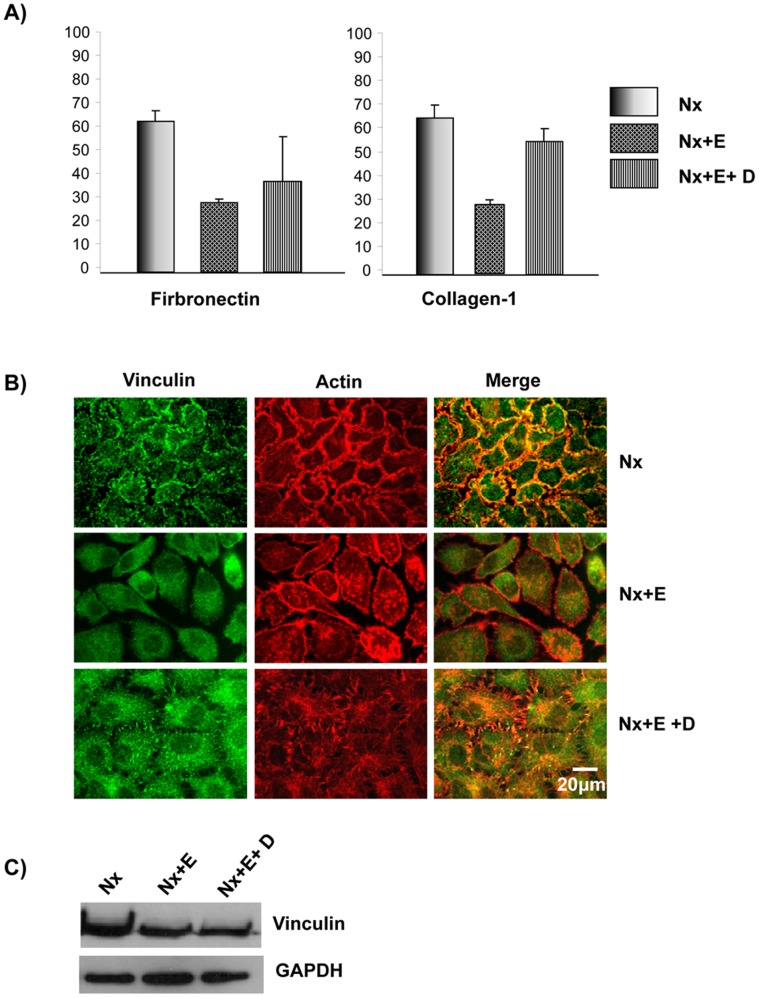
EGF treatment reduced cell-ECM adhesion. A) Cell-ECM adhesion is diminished in cells treated with EGF. A431 cells were grown under normoxia (Nx), normoxia+EGF (Nx+E) or normoxia+EGF+Cetuximab (Nx+E+D). The cells were used to carry out cell adhesion assay as described in the Materials and Methods. B) EGF treatment led to loss of vinculin patches. A431 cells were grown as in Panel A and probed with anti-vinculin antibody followed by Alexa488 secondary antibody. The actin cytoskeleton was visualized using Alexa568-Phalloidin. C) EGF treatment does not alter the expression of vinculin. A431 cells were grown under normoxia, normoxia+EGF or normoxia+EGF+Cetuximab. Cell lysate were analyzed by immunoblotting with anti-vinculin or anti-GAPDH primary antibodies.

### Immunofluorescence

Cells grown on coverslips were fixed with 4% formaldehyde in PBS for 15 minutes, permeabilized with 0.4% Triton X-100 in PBS for 30 min and blocked with 1% BSA in PBS for 30 min. The cells were incubated with appropriate Mouse antibodies diluted (1∶50) in blocking solution and incubated for 1 hr. The cells were washed with PBS and incubated with Alexa488-conjugated goat anti-mouse antibody for 1 hr. The cells were washed with PBS and stained with Alexa568-Phalloidin. Fluorescent images were taken with Olympus microscope fitted with Photo metrics Cool Snap HQ2 camera.

**Figure 8:**
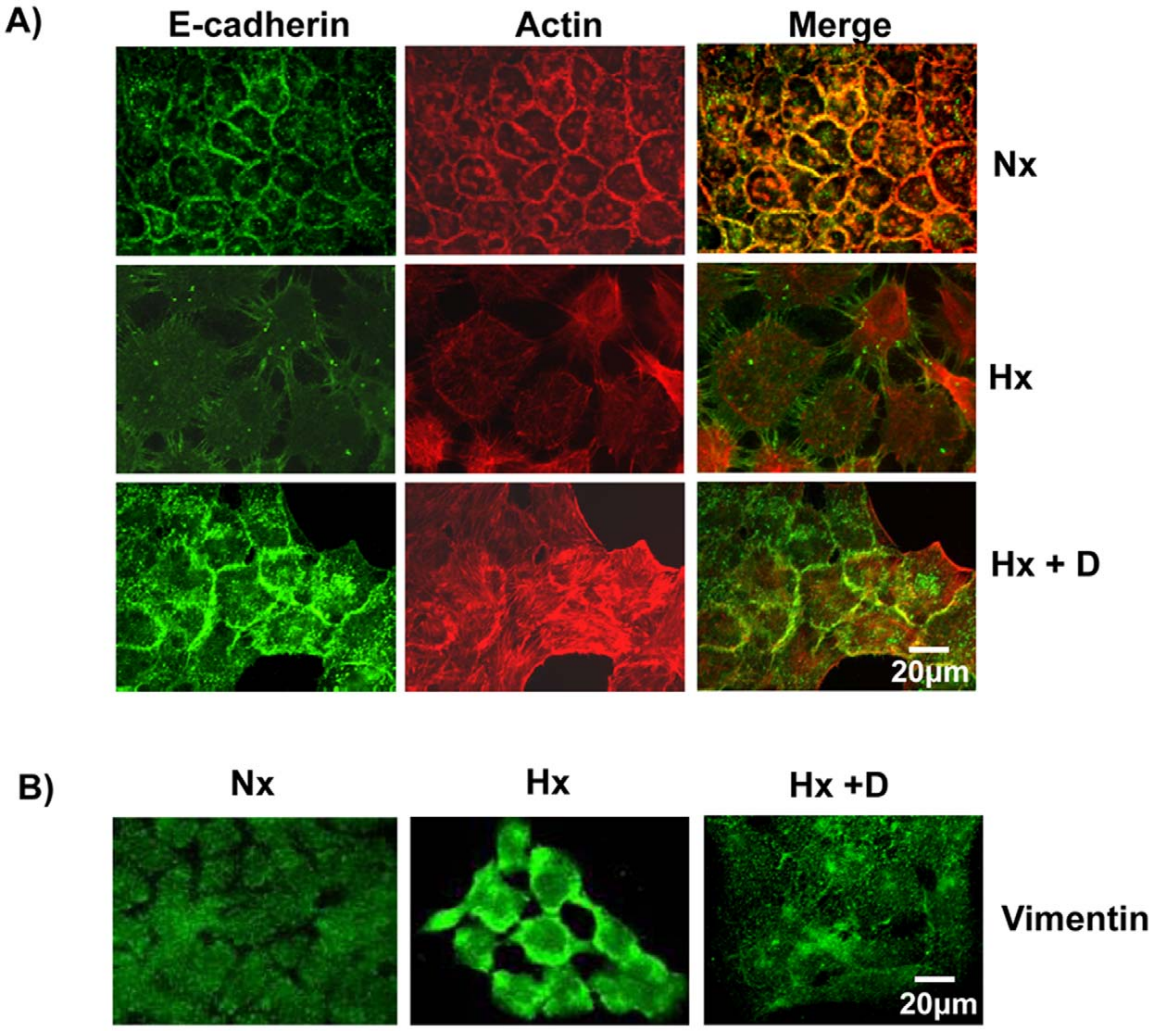
Cetuximab blocked hypoxia-induced EMT. A) Cetuximab blocked hypoxia-induced loss of cell-cell contacts. A431 cells were grown under normoxia, hypoxia or hypoxia+Cetuximab. The cells were fixed, permeabilized and probed with anti-E-cadherin primary antibodies followed by labeled secondary antibodies. The actin cytoskeleton was visualized using Alexa568-Phalloidin. B) Cetuximab blocked hypoxia-induced expression of vimentin. A431 cells were grown under normoxia, hypoxia or hypoxia+Cetuximab. The cells were fixed, permeabilized and probed with anti-vimentin primary antibodies followed by labeled secondary antibodies.

**Figure 9:**
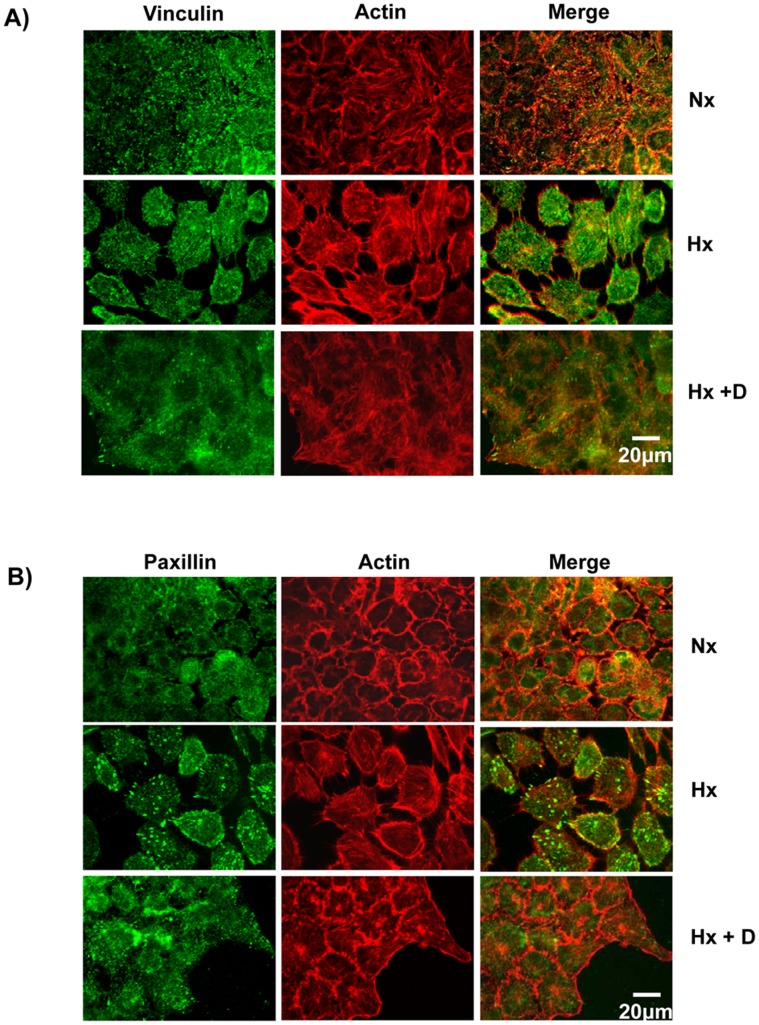
Cetuximab blocked hypoxia-induced redistribution of vinculin and paxillin. A431 cells were grown under normoxia, hypoxia or hypoxia plus Cetuximab conditions and probed with either anti-vinculin (A) or anti-paxillin (B) followed by labeled secondary antibodies. The actin cytoskeleton was visualized using Alexa568-Phalloidin.

### Statistical Analysis

Statistical significance analysis was performed using one-way Anova or student t-test and P<0.05 was considered statistically significant. Values presented in bar charts represent mean±S.D of at least three independent experiments.

## Results

### Growth Under Hypoxia Conditions Lead to Loss of Cell-cell Contact

E-cadherin, a transmembrane adhesion molecule which localizes predominantly at the apical and lateral membrane junction mediates cell-cell adhesion [20]. The extra-cellular domains of E-cadherin molecules on adjacent cells bind through homotypic interactions while the intracellular domains bind to the actin cytoskeleton through the adaptor proteins such as α-catenin and β-catenin [21]. Thus E-cadherins are essential for the formation of epithelial sheets in metazoan. Cell migration is central to development and tissue remodeling and has a major role in cancer and metastasis [22]. The loss of cell-cell adhesion formed by E-cadherin is one of the hallmarks of EMT and is an essential step in metastasis. In order to elucidate the molecular mechanisms involved in hypoxia-induced EMT, we grew A431 cells under normoxic conditions (21% O_2_, 5% CO_2_) or under hypoxia conditions (95% N_2_, 5% CO_2_) for 3 days and analyzed the expression of E-cadherin by both western blot and localization by immunofluorescence. The cells grown under normoxia conditions formed sheet-like structures with E-cadherin localized at the cell boundary while the cells grown under hypoxia conditions did not form sheet-like structures (Fig. 1A). E-cadherin in the cells grown under normoxia conditions co-localized with the actin cytoskeleton (Fig. 1A). Lack of E-cadherin at the cell boundary in cells grown under hypoxic conditions was due to reduced expression of E-Cadherin as shown by western blot analysis (Fig. 1B). The expression of E-cadherin was restored when the cells grown in hypoxia conditions were then grown under normoxia conditions (Fig. 1B). We also analyzed the expression of Vimentin, a mesenchymal marker and found that cells grown under normoxia conditions did not express Vimentin, while cells grown in hypoxia conditions expressed high levels of Vimentin (Fig. 1C). The cells grown in hypoxia conditions, upon returning to normal oxygen levels, lost the expression of Vimentin (Fig. 1C). This suggests that epithelial cells grown under normoxic conditions express E-cadherin but do not express Vimentin while growing the cells under hypoxic conditions led to down regulation of E-cadherin and up regulation of Vimentin expression and this was a reversible process as returning the epithelial cells grown in hypoxic conditions to normoxic conditions led to up regulation of E-cadherin expression and down regulation of Vimentin expression (Fig. 1B, C).

### Growth Under Hypoxia Conditions Led to Increased Cell Motility

Metastasis involves the migration of cells away from the primary tumor site to secondary organs and establishment of secondary tumors, thus cell migration has a major role in cancer metastasis [22]. We therefore analyzed the cell migration characteristics of epithelial cells grown under normoxic and hypoxic conditions. Cells grown under normoxia as well as hypoxia for 3 days were seeded in 6 wells culture plate and allowed to grow to confluence under the same growth conditions. A scratch was made in the middle of the culture well using a yellow micropipette tip and images were acquired before incubating the cells for 16 hours at 37°C. The image of the scratch was also acquired after 16 hours (Fig. 2A) which showed that cells grown under hypoxic conditions filled the scratch more efficiently than the cells grown under normoxia or cells grown under hypoxia followed by growth under normoxia conditions (Hx+Nx). This suggests that growth under hypoxia leads to increased cell motility.

Rac1 (Ras-related C3 botulinum toxin substrate 1), is a small GTPase involved in signal transduction and has been shown to be involved in the regulation of many cellular processes including cell-cell adhesion and cell motility [23]. Thus, we analyzed the expression and localization of Rac1 in cells grown in hypoxia conditions. Immunostaining for Rac1 in cells grown under normoxia conditions did not show any predominant localization of Rac1 as it was distributed throughout the cell (Fig. 2B). However, cells grown under hypoxic conditions showed a predominant plasma membrane localization of Rac1 which partially co-localized with the actin cytoskeleton (Fig. 2B). This suggests that growth under hypoxic conditions led to localization of Rac1 to the plasma membrane. Rac1 was expressed at comparable levels in both the cells grown under normoxic and hypoxic conditions (Fig. 2C), suggesting that Rac1 expression is not altered by growth in hypoxia. Thus, the predominant plasma membrane localization of Rac1 is due to relocalization of Rac1. At the plasma membrane, Rac1 is known to function as a molecular switch and regulate the activity of proteins such as WAVE2 which activate the Arp2/3 complex and promote cell motility [24].

### Hypoxia Leads to Reduced Cell-ecm Adhesion and Spreading

We have previously found a correlation between increased motility and reduced cell-ecm adhesion [25]. Thus, we analyzed the cell-ecm adhesion properties of A431 cells grown under normoxia and hypoxia conditions using fibronectin and collagen-I as substrate. Cells grown under normoxia conditions were found to adhere to both fibronectin and collagen-I at comparable levels but did not adhere to BSA (Fig. 3A) while cells grown under hypoxia conditions exhibited a reduced adhesion to both fibronectin and collagen-I suggesting that hypoxia affected the cell-ecm adhesion properties of the cells (Fig. 3A). We also analyzed the spreading ability of cells grown under normoxia and hypoxia conditions using fibronectin or collagen-I coated wells (Fig. 3B, C). The images showed that cells grown under hypoxia took a longer time to spread compared to cells grown under normoxia conditions. The hypoxia-induced reduction in cell-ecm adhesion was a reversible process as cells grown in hypoxia conditions, when returned to normoxic conditions, exhibited normal cell-ECM adhesion and spreading.

### Hypoxia Led to Redistribution of Vinculin and Paxillin

Epithelial cells are held in place by both cell-cell and cell-ECM adhesion. While the cell-cell adhesion is mediated by E-cadherin, cell-ECM contacts are mediated through integrin-ECM binding at focal adhesion sites made up of at least 50 different proteins including vinculin and paxillin [26]. We have previously found that cells with increased motility had reduced vinculin patches [25], [27]. In cells grown under normoxic conditions, well defined vinculin patches were seen in the cell periphery and these patches co-localized partially with the actin cytoskeleton (Fig. 4A). Growth of the cells under hypoxic conditions led to the loss of the vinculin patches from the cell periphery and this was not due to reduced expression of vinculin (Fig. 4C). The loss of vinculin patches was a reversible process as growing the cells in normal conditions after growth in hypoxia resulted in the reappearance of vinculin patches (Fig. 4A). The localization of paxillin was opposite to that of vinculin, paxillin patches were not visible when the cells were grown under normoxic conditions (Fig. 4B) and this was not due to lack of expression of Paxillin (Fig. 4C). Growth of A431 cells under hypoxic conditions resulted in well defined paxillin patches at the cell periphery (Fig. 4B) which was not due to increased expression. The paxillin patches were drastically reduced when the cells grown in hypoxia conditions were re-grown under normoxic conditions. Thus, our results suggest that cells grown under normoxic conditions are less motile probably due to the presence of well defined vinculin patches which help to anchor the cells while cells grown under hypoxic conditions are highly motile due a to reduced number of vinculin patches.

### Hypoxia Induces Increased Expression of EGFR

Over expression of EGFR and altered EGF signaling are common features in a variety of human cancers [28], [29]. A431 cells are known to be sensitive to EGF probably due to the high level of expression of EGFR [30] and it has been suggested that hypoxia induced translational upregulation of EGFR [31]. EGF has been identified as a novel EMT inducer in human breast cancer [32]. Thus we analyzed the expression of EGFR in cells grown under various growth conditions using two antibodies specific for the intracellular domain and extracellular domain of EGFR (Fig. 5A). Cells grown under normal oxygen conditions expressed very low levels of EGFR and this expression was enhanced by growth of the cells under hypoxic conditions (Fig. 5A). EGFR has been shown to associate with N-WASP a known effector of Rac1 [33]. Thus we analyzed the expression of N-WASP (Fig. 5B) and found that the expression of N-WASP is upregulated under Hypoxic conditions. This suggests that the enhanced membrane localization of Rac1 (Fig. 2B) coupled with increased N-WASP expression may lead to the increased cell motility under EMT.

### EGF Stimulation Leads to Loss of Cell-cell Contacts and Decreased Cell-ECM Adhesion

EGF is known to promote EMT [32]. We therefore analyzed the effect of EGF treatment on A431 cells. Growth of A431 cells under normoxic conditions with 50 ng/ml of EGF (Nx+E) led to the loss of cell-cell contacts as displayed by the loss of E-cadherin at the cell boundaries, and this was due to loss of E-cadherin expression (Fig. 6A, B). Treatment of A431 cells with EGF also led to increased expression of vimentin (Fig. 6B). We determined the cell-ECM adhesion characteristics of EGF-treated A431 cells as described in the Material and Methods. The untreated cells adhered to both fibronectin and collagen-I, while the EGF-treated cells displayed a reduced cell-ECM adhesion (Fig. 7A). We also analyzed the focal adhesion complex by staining for vinculin. While the untreated cells were found to have well defined vinculin patches, the EGF-treated cells showed a vinculin staining pattern (Fig. 7B) similar to that found in cells grown under hypoxia conditions (Fig. 4A) and this is not due to changes in the expression of vinculin (Fig. 7C). We tested the specificity of the EGF treatment using the drug Cetuximab. Cetuximab is a human-mouse chimeric monoclonal antibody that acts as EGFR inhibitor [34]. It binds to the extracellular domain of EGFR and prevents activation of EGF signaling pathway. Treatment of A431 cells with Cetuximab abolished the effects of EGF (Fig. 6 and 7). The cells treated with the drug alone had no effect in terms of vinculin staining or cell-ECM adhesion (data not shown).

### Cetuximab Blocked HYPOXIA Induced EMT

Hypoxia induced the upregulation of EGFR expression (Fig. 5), thus we analyzed the effect of Cetuximab on the hypoxia-induced EGFR expression. Cetuximab abolished EGF-induced expression of EGFR as well as the hypoxia-induced expression of EGFR (Fig. 5). We subsequently analyzed the effect of Cetuximab on the other hypoxia-induced phenotypes. A431 cells grown under hypoxia conditions in the presence of Cetuximab still expressed E-cadherin (Fig. 1B) and the E-cadherin was localized in the cell boundary and co-localized partially with the actin cytoskeleton (Fig. 8A). A431 cells grown under hypoxia in the presence of Cetuximab did not express Vimentin (Fig. 1C, Fig. 8B). Hypoxia did not affect the expression of vinculin or paxillin (Fig. 4C) but affected the distribution of vinculin and paxillin patches (Fig. 4A, B). Addition of Cetuximab during growth in hypoxia abolished the re-distribution of vinculin and paxillin (Fig. 9A, B). Thus, Cetuximab blocked all the changes induced by hypoxia suggesting that the hypoxia-induced phenotypes are mediated through increased expression of EGFR and the activation of EGFR signaling.

## Discussion

Metastasis is a multistep process which involves destruction of the basement membrane and local invasion at the primary site, intravasation and survival in the circulatory system, extravasation into distant sites and survival plus proliferation at the secondary site [35]. Most human cancers originate from epithelial cells [1], which undergo EMT before metastasis [9]. EMT involves a number of molecular changes; loss of cell-cell contact, remodeling of the actin cytoskeleton, loss of polarity, induction of mesenchymal-specific gene expression and migration through the basement membrane. Thus, EMT does not denote a single molecular event, but a number of events which change the epithelial cells to be more like mesenchymal cells [36]. It is now well established that EMT plays a significant role in metastasis [37] and renal fibrosis [38].

Growth of solid tumors is limited by the availability of oxygen which is in turn limited by the extent of vascularization. It has been suggested that hypoxia develops within 100–200 µm from blood vessels [39]. While most cancer cells die due to poor adaptation to hypoxia, some cancer cells may undergo adaptations which include changes in signaling and gene expression which promote cell survival and metastasis [40]. Thus, it is crucial to characterize the effects of hypoxia on cell physiology. Characterization of the molecular mechanisms involved in the adaptive mechanisms employed by cancer cells under hypoxia will lead to the identification of molecules which may be targeted to control the effects of hypoxia on metastasis.

In this study, we have shown that hypoxia induces cellular transitions in A431 cells. A431 cells grown under hypoxia failed to form an epithelial sheet, unlike cells grown under normal O_2_ tension (Fig. 1A). This is probably due to the down regulation of E-cadherin expression in cells grown under hypoxia. Cells grown under hypoxia adhered poorly to ECM proteins collagen-I and fibronectin compared to the controls grown under normal oxygen tension (Fig. 3A). Similarly the cells grown in hypoxia spread slowly on surfaces coated with ECM proteins suggesting differences in the adhesion characteristics of cells induced by hypoxia. Cells grown under hypoxia showed increased motility as well (Fig. 2A), probably due to poor cell-ECM adhesion. The two focal adhesion proteins vinculin and paxillin also showed differences in their localization patterns (Fig. 4). Cells grown under normal conditions had well defined vinculin patches which co-localized with the actin cytoskeleton, while cells grown under hypoxia had very small vinculin patches (Fig. 4A). Paxillin staining showed the opposite phenomenon, cells grown under normal conditions had very small paxillin patches while cells grown under hypoxia had well defined patches at the cell periphery (Fig. 4B). Vinculin^−/−^ fibroblast do assemble focal adhesions, however they spread slowly on surfaces coated with ECM proteins and migrate faster in a wound healing assay compared to the Vinculin^+/+^ fibroblast [41]. This suggests that though hypoxia does not affect the expression of vinculin (Fig. 4C) it affects the recruitment of vinculin to patches leading to poor adhesion, spreading and increased motility. This is consistent with our previous finding that loss of N-WASP leads to reduced Vinculin patches, reduced adhesion and increased cell motility [25]. Paxillin expression is elevated in metastatic cells and knocking down paxillin expression lead to reduced cell motility [42]. Thus, hypoxia leads to the formation of prominent paxillin patches probably due to increased recruitment of paxillin rather than increased expression (Fig. 4C). Hypoxia also induced recruitment to the plasma membrane of Rac1, a protein which promotes cell motility [23]. At the plasma membrane, Rac1 is known to function as a molecular switch and regulate the activity of proteins such as WAVE2 and N-WASP which activate the Arp2/3 complex and promote cell motility [24], [33]. Hypoxia was also found to upregulate N-WASP expression (Fig. 5B) and N-WASP has been shown to be activated by EGFR [43] and Rac1 [33].

Hypoxia leads to increased expression of EGFR (Fig. 5A) and addition of EGF results in increased expression of EGFR and phenotypes similar to that induced by hypoxia, loss of E-cadherin expression, increased expression of vimentin, and poor adhesion (Fig. 6 and 7). EGF promotes cell proliferation [44], cell differentiation [45] as well as cell motility [46], [47]. The EGF-induced EMT phenotypes were abolished by Cetuximab, a monoclonal antibody which blocks EGF signaling by binding to EGFR. Cetuximab also blocked the hypoxia-induced EMT suggesting that hypoxia promotes EMT by upregulating EGF signaling through increased expression of EGFR. A recent study has implicated PI3 kinase/Akt signaling in hypoxia-induced EMT in hepatocellular carcinoma cells [16] and EGFR is required for the Akt activation [48].

We have shown that hypoxia induces EMT by upregulating EGF signaling which leads to alteration in the expression of genes E-cadherin (reduced expression) and vimentin (increased expression). Hypoxia also altered the localization of cell-ECM adhesion proteins (vinculin, paxillin) as well as increased localization of Rac1 to the plasma membrane leading to changes in the cell-ECM adhesion and increased cell motility characteristics of EMT.
